# An unusual tumor mimicker in the iliac wing: A hydatid cyst

**DOI:** 10.1590/0037-8682-0344-2022

**Published:** 2022-09-30

**Authors:** Nurmuhammet Tas, Kutsi Tuncer, Yener Aydin

**Affiliations:** 1 Erzurum Regional Education and Research Hospital, Department of Physical Medicine Rehabilitation and Rheumatology, Erzurum, Turkey.; 2 Altıntas University, Medical Faculty, Department of Orthopedics and Traumatology, Istanbul, Turkey.; 3 Ataturk University, Medical Faculty, Department of Thoracic Surgery, Erzurum, Turkey.

**FIGURE 1:**
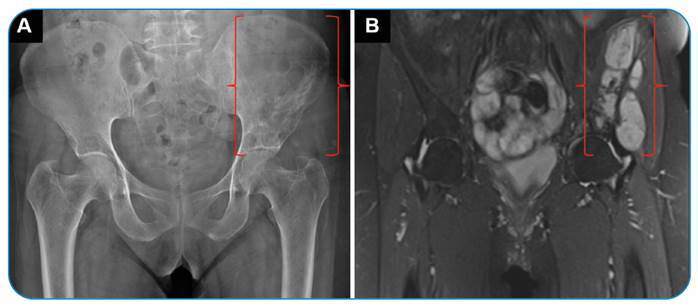
Direct radiography **(A)** shows a large expansile lytic lesion (in brackets) in the left iliac wing. Magnetic resonance images **(B)** reveal multicystic iliac lesions (in brackets).

A 49-year-old woman presented with left hip pain and movement limitation. Direct radiography showed a large expansile lytic lesion without cortical destruction in the left iliac wing. Magnetic resonance imaging revealed a multicystic left iliac lesion (Figure 1). Histopathologic examination revealed a hydatid cyst. 

Hydatid cysts caused by the larval stage of *Echinococcus granulosus* remain an important health problem^1^. The disease is primarily located in the liver and lungs; involvement of the musculoskeletal system is rare, especially bone localization, which occurs in less than 1% of cases^2^. The spine is most commonly involved in bone structure in such cases^3^. The treatment of bone-located hydatid cysts is more difficult compared to other regions^2^ ^,^ ^3^. Although rare, bone hydatid cyst is a clinically serious disease. Destructive growth in hydatid cysts can lead to high rates of morbidity similar to locally malignant bone tumors^3^. Iliac involvement is relatively rare and can mimic cystic neoplasms of the iliac bone. 
